# Mitochondrial Neurodegenerative Diseases: Three Mitochondrial Ribosomal Proteins as Intermediate Stage in the Pathway That Associates Damaged Genes with Alzheimer’s and Parkinson’s

**DOI:** 10.3390/biology12070972

**Published:** 2023-07-08

**Authors:** Luigi Del Giudice, Paola Pontieri, Mariarosaria Aletta, Matteo Calcagnile

**Affiliations:** 1 Istituto di Bioscienze e BioRisorse-UOS Napoli-CNR c/o Dipartimento di Biologia, Sezione di Igiene, 80134 Napoli, Italy; 2 DCSRSI SPR BIBLIOTECA, 80131 Napoli, Italy; 3 Dipartimento di Scienze e Tecnologie Biologiche e Ambientali, Università del Salento, 73100 Lecce, Italy; matteo.calcagnile@unisalento.it

**Keywords:** mitochondrial neurodegenerative diseases, mitochondrial ribosomal proteins, MRPL44, NAM9, GEP3, Alzheimer’s disease, Parkinson’s disease

## Abstract

**Simple Summary:**

Neurodegenerative diseases are characterized by the progressive degeneration of nerve cells. Some neurodegenerative diseases such as Alzheimer’s and Parkinson’s are caused by disorders in the mitochondria, which are organelles present in the eukaryotic cells of animals, plants and fungi, and their function is to produce energy. The formation and function of mitochondria are influenced by two separate genetic inheritances physically and functionally present in the cell: nuclear DNA, which provides 90% of key components such as proteins, and mitochondrial DNA, which provides 5% of these components. Recently, three genes in the nuclear DNA have been identified that encode for three mitochondrial ribosomal proteins, namely MRPL44, NAM9 and GEP3, respectively. The absence of or defects in these proteins could potentially be the cause of neurodegenerative diseases such as Alzheimer’s and Parkinson’s. In this review, we present the recent advances in the most common mitochondrial neurodegenerative diseases, the mitochondrial ribosomal proteins associated with mitochondrial neurodegenerative diseases and the aforementioned three ribosomal proteins. Finally, we evaluate our experimental hypothesis useful for the characterization of these three ribosomal proteins in order to understand their role in mitochondrial neurodegenerative dysfunctions, which could lead to the development of potential therapeutic drugs for the treatment of such diseases.

**Abstract:**

Currently, numerous research endeavors are dedicated to unraveling the intricate nature of neurodegenerative diseases. These conditions are characterized by the gradual and progressive impairment of specific neuronal systems that exhibit anatomical or physiological connections. In particular, in the last twenty years, remarkable efforts have been made to elucidate neurodegenerative disorders such as Alzheimer′s disease and Parkinson′s disease. However, despite extensive research endeavors, no cure or effective treatment has been discovered thus far. With the emergence of studies shedding light on the contribution of mitochondria to the onset and advancement of mitochondrial neurodegenerative disorders, researchers are now directing their investigations toward the development of therapies. These therapies include molecules designed to protect mitochondria and neurons from the detrimental effects of aging, as well as mutant proteins. Our objective is to discuss and evaluate the recent discovery of three mitochondrial ribosomal proteins linked to Alzheimer′s and Parkinson′s diseases. These proteins represent an intermediate stage in the pathway connecting damaged genes to the two mitochondrial neurological pathologies. This discovery potentially could open new avenues for the production of medicinal substances with curative potential for the treatment of these diseases.

## 1. Introduction

**Figure 1:**
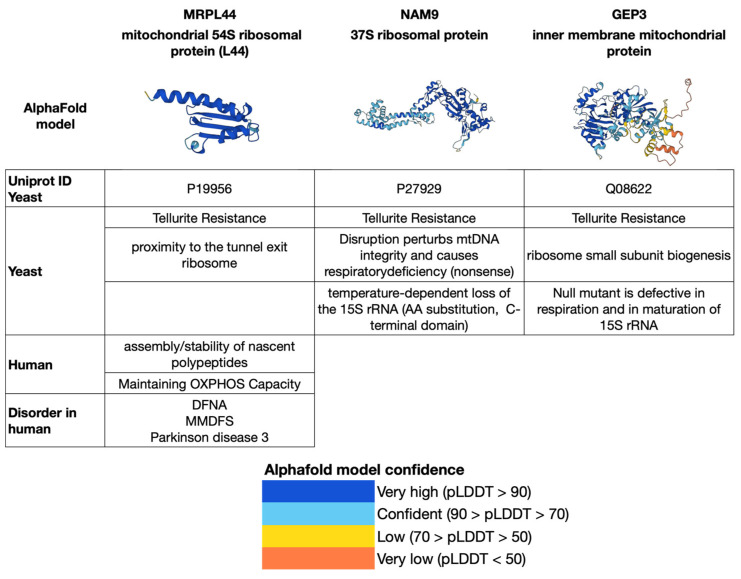
Diagram showing the 3D structure generated by AlphaFold and the effect of mutations in yeast of the two mitochondrial ribosomal proteins (MRPL44 and NAM9) and the mitochondrial biogenesis ribosomal protein (GEP). The relationship with some degenerative diseases in humans is also shown for the MRPL44 protein.

Luft et al. published in 1962 the first case of mitochondrial dysfunction in a 35-year-old woman affected by myopathy [1]. Since then, there has been a continuous advancement of research that is leading to the understanding, from a medical perspective, of the role of mitochondria in health, diseases and aging. Many seemingly unrelated pathologies, such as neuropathies, Alzheimer’s, Parkinson’s, myopathies, ataxia, cancer and others, share common underlying pathophysiological procedures. These mechanisms include the production of reactive oxygen species (ROS) and the damage they cause to the mitochondrial genome, leading to mitochondrial dysfunction [2,3,4]. As mitochondrial formation arises from the contribution of two distinct genomes, namely the nuclear DNA (nDNA) and the mitochondrial DNA (mtDNA) [5,6,7], genetic mutations in either the nDNA or the mtDNA, as well as genetic deletions in the mtDNA, can give rise to mitochondrial disease. These genetic mutations specifically result in defects in the mitochondrial oxidative phosphorylation system (OXPHOS) [8], which are the underlying cause of mitochondrial diseases [9,10,11]. Extensive research has been conducted on mutations occurring in nuclear genes associated with mitochondrial disorders, specifically focusing on their connection to diseases associated with premature aging [12,13] and POLG-related disorders that exhibit neurological symptoms [3,14,15]. Mitochondrial diseases represent the most common group of inherited metabolic dysfunction and are among the most prevalent types of inherited neurological disorders [16]. Mitochondrial disorders resulting from recurrent mutations in mtDNA manifest as shared syndromes across unrelated families and populations [2,17]. These disorders exhibit a wide range of clinical variations and can emerge at any age [16]. Given that mitochondria exist in all cells of the body except for red blood cells, the resulting clinical symptoms may manifest in specific organs independently but frequently involve multiple systems in organs that have high energy requirements, such as the brain, skeletal system, muscles and heart [16]. With reference to mutations in mtDNA or nDNA, from the point of view of clinical diagnosis, primary mitochondrial disease (PMD) and secondary mitochondrial dysfunction (SMD) are distinguished [18,19]. The primary differentiation between PMD and SMD lies in the fact that PMD genes either directly encode OXPHOS proteins or influence OXPHOS function by affecting the production of the complex machinery required for the OXPHOS process. On the other hand, SMD can be caused by genes that do not encode OXPHOS proteins or affect their production. SMD is often associated with various hereditary nonmitochondrial diseases [11,18,19]. There are several factors that determine the onset of mitochondrial dysfunction such as age, heredity factors, an unhealthy lifestyle or stress, environmental toxicity and more [7,16,20,21,22,23,24]. Among the mitochondrial diseases, the most widely known are neurodegenerative diseases, which are mainly caused by genetic factors and by environmental factors [7,24,25]. Neurodegenerative diseases are stress-inducing brain disorders characterized by behavioral, motor and cognitive impairments. Numerous studies conducted over the years have provided evidence that mitochondrial dysfunction serves as the underlying cause in the development of neurodegenerative diseases, which include Alzheimer’s disease (AD), Parkinson’s disease (PD), Huntington’s disease (HD), Amyotrophic Lateral Sclerosis (ALS) and Dementia [26,27,28,29]. These diseases arise from deficiencies in mitochondrial function, which is primarily regulated by over 1000 proteins encoded by both the mitochondrial and nuclear genomes [30]. Studies have shown that the majority, around 90%, of mitochondrial proteins are encoded by genes found in the cell nucleus. These proteins are synthesized by ribosomes in the cytosol and subsequently transported into mitochondria through a complex import machinery composed of multiple components [5,6,31,32,33,34]. These proteins have various functions attributed to them, including respiration, metabolite transport, protein translocation, redox homeostasis and other processes that are interconnected within complex and dynamic networks. The malfunctioning of these systems could lead to the development of diseases [35]. Despite mitochondria having their own genome, many nuclear-encoded mitochondrial ribosomal proteins (MRPs) are essential for proper organelle function. Mutations in Mrp genes, as indispensable components of the mitochondrial translation machinery, are detrimental to the OXPHOS system and associated with several neurodegenerative diseases in humans [36,37,38,39]. In a recent study using the yeast *Saccharomyces cerevisiae* (*S. cerevisiae*) as a model for the study of neurodegenerative diseases in humans, three nuclear genes, MRPL44, NAM9 (MNA6) and GEP3 (MTG3), were identified to encode three ribosomal mitochondrial proteins (Figure 1). The absence of or defects in these proteins was associated with neurodegenerative diseases in humans, such as AD and PD [40,41]. In the following paragraphs, we will provide a brief review of the recent progress made in understanding mitochondrial neurodegenerative diseases, specifically focusing on mitochondrial ribosomal proteins. We will discuss three MRPs that have been identified as intermediate stages in a pathway associated with AD and PD. Additionally, we will present an experimental hypothesis regarding the isolation and characterization of three specific mitochondrial ribosomal proteins: MRPL44, NAM9 and GEP3 (Figure 1). These proteins have been found to be connected with neurodegenerative diseases.

## 2. Mitochondrial Neurodegenerative Diseases

**Figure 2:**
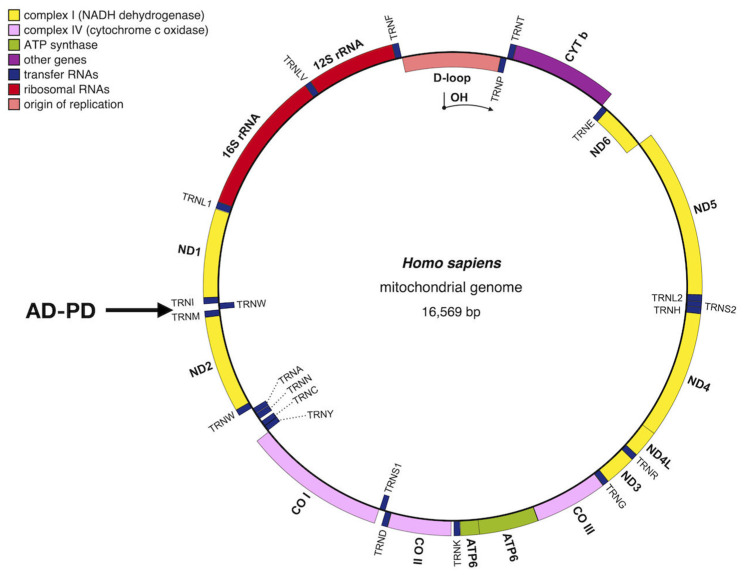
Schematic map of the human mitochondrial genome with related neurodegeneration diseases (adapted with minor modification from [2,19], CO: cytochrome oxidase (COX) subunit genes; CYTb = cytochrome b; ND = OXPHOS complex I subunit genes; D-loop = noncoding displacement loop or control region; AD-PD = neurodegenerative diseases: Alzheimer’s disease and Parkinson’s disease. Image generated with OGDRAW [83].

The human mitochondrial genome consists of a circular molecular structure comprising approximately 16,569 pairs of nucleotides (Figure 2). It contains a total of 24 mtDNA genes, including the small ribosomal RNA (12S rRNA) and large ribosomal RNA (16S rRNA) genes, as well as 22 transfer RNA (tRNA) genes necessary for translating the 13 respiratory chain proteins [19,41,42].

Studies conducted over the past forty years have highlighted the role of mitochondria in both normal brain function and the pathogenesis of diseases originating from them (Table 1) [43]. Mitochondrial diseases result from hereditary or spontaneous mutations in mtDNA or nDNA, leading to abnormalities in the functions of proteins or RNA molecules typically found within mitochondria [41,44,45]. Generally, the term “mitochondrial disease” describes a heterogeneous set of conditions caused by genetic defects in the assembly and/or function of OXPHOS proteins [45]. In fact, mitochondria play a fundamental role in energy production and are essential in numerous cellular processes [35,46]. Consequently, mitochondrial dysfunction often propagates and/or underlies many pathological states, including neurodegenerative diseases [47,48] and the aging process [49,50]. More recently, extensive research efforts have helped define some of the pathological mechanisms underlying neurodegenerative processes, such as the two most common neurodegenerative disorders: AD and PD.

**Table 1:** Mitochondrial diseases ^a^.

Mitochondrial Pathology	mtDNA Mutations	Clinical Syndromes	References
CPEO	single deletion	Loss of the muscle functions involved in eye and eyelid movement	[51,52]
KSS	single deletion	Neuromuscular disorder	[53,54]
PS	single deletion	It affects various parts of the body, especially bone marrow and the pancreas	[55,56]
Diabetes and deafness	single deletion	Hyperglycemia and reduction or absence of hearing ability	[57,58]
Encephalomyopathy	multiple deletions	Muscle weakness and pain, recurrent headaches, loss of appetite, vomiting and seizures	[59,60]
Recurrent myoglobinuria	multiple deletions	Metabolic disturbances that include hypokalemia, hypophosphatemia, hyponatremia, hypocalcemia and hypernatremia	[61,62]
SANDO	multiple deletions	Impaired coordination (ataxia), slurred speech (dysarthria) and weakness of the eye muscles (ophthalmoparesis)	[63,64]
LHON	point mutation	Progressive visual loss due to optic neuropathy	[65,66]
MELAS	point mutation	Disease primarily affecting the nervous system and muscles	[67,68]
NARP	point mutation	Neurogenic muscle weakness, sensory-motor neuropathy, ataxia and pigmentary retinopathy	[69,70]
MERRF	point mutation	Progressive myoclonus and seizures, cerebellar ataxia, myopathy, cardiac arrhythmia, sensorineural hearing loss, optic atrophy and dementia	[71,72]
CPEO	point mutation	Loss of the muscle functions involved in eye and eyelid movement	[19,73]
Leigh syndrome	point mutation	Neurological disorder involving elevated blood and/or cerebrospinal fluid levels of lactate, developmental retardation, hypotonia, followed by respiratory dysfunction, epileptic seizures, poor feeding and weakness	[74,75]
AD	nuclear gene mutation	Brain disorder that slowly destroys memory and thinking skills and, eventually, the ability to carry out the simplest tasks	[41,76]
PD	nuclear gene mutation	Combinations of motor problems—namely, bradykinesia, resting tremor, rigidity, flexed posture, “freezing,” and loss of postural reflexes	[76,77]
FRDA	nuclear gene mutation	Progressive ataxia, absent lower limb reflexes, upgoing plantar responses and peripheral sensory neuropathy.	[76,78]
HD	nuclear gene mutation	Disorder that causes nerve cells (neurons) in parts of the brain to gradually break down and die	[76,79]
ALS	nuclear gene mutation	Progressive nervous system disease that affects nerve cells in the brain and spinal cord, causing loss of muscle control	[76,80]
HSP	nuclear gene mutation	Disorder that causes the small blood vessels in skin, joints, intestines and kidneys to become inflamed and bleed	[76,81]
Aging	nuclear gene mutation	Accumulation of biological changes leading to functional decrease in the organism	[76,82]

^a^ Adapted with minor modification from Chinnery et al. [44]. CPEO = chronic progressive external ophthalmoplegia; KSS = Kearns-Sayre syndrome; PS = Pearson syndrome; SANDO = sensory ataxic neuropathy, dysarthria and ophthalmoparesis; LHON = Leber hereditary optic neuropathy; MELAS = mitochondrial encephalopathy, lactic acidosis and stroke-like episodes; NARP = neuropathy, ataxia and retinitis pigmentosa; MERRF = myoclonus epilepsy and ragged red fiber disease; AD = Alzheimer’s disease; PD = Parkinson’s disease; FRDA = Friedreich’s ataxia; HD = Huntington’s disease; ALS = amyotrophic lateral sclerosis; HSP = Henoch-Schönlein purpura.

### 2.1. Alzheimer’s Disease in Brief

AD, a neurodegenerative condition linked to the aging process, is characterized by progressive cognitive and memory decline. It stands as the most prevalent neurodegenerative disorder, comprising around 60–70% of all dementia cases and impacting approximately 6% of individuals aged 65 and above (referred to as late-onset AD). Furthermore, a smaller subset of patients, around 2–10%, experience early-onset AD [41,84,85]. At the cellular level, AD is characterized by progressive and irreversible deterioration of neuronal structure and function in specific brain regions, including the hippocampus and neocortical areas, leading to cognitive dysfunction and dementia [86]. The main neuropathological features of the disease include the presence of extracellular β-amyloid plaques and intracellular neurofibrillary tangles formed by hyperphosphorylated tau proteins [87,88,89]. The causal relationship between the presence of these two hallmarks and AD is still not fully understood. It remains uncertain whether they are the primary cause of the disease or predominantly the outcome of a cascade of cellular events, including oxidative stress, mitochondrial dysfunction and apoptosis. Nonetheless, the exact mechanism by which these proteins damage neurons is still unknown [88,90]. Despite AD being the leading cause of dementia worldwide and the growing population of AD patients, no new therapies have received approval for over a decade [91,92].

### 2.2. Parkinson’s Disease in Brief

PD is the most common movement disorder and the second most frequent age-related neurodegenerative disorder in the world after AD [93,94]. It is a progressive and irreversible pathology characterized by significant neuronal loss in the pars compacta of the substantia nigra, leading to an alteration in dopaminergic transmission [95,96] and the presence of intracellular inclusions called Lewy bodies, which contain aggregates of α-synuclein [97,98]. The disease’s etiology is attributed to a combination of genetic and environmental factors [97]. In large populations, 5–10% of PD cases are attributed to known PD genes, indicating monogenic PD. However, 90 genetic risk variants collectively contribute only 16–36% to the genetic risk of nonmonogenic PD [94]. From a genetic standpoint, definitively characterized PD genes include autosomal dominant forms (SNCA, LRRK2 and VPS35) and autosomal recessive forms (PRKN, PINK1 and DJ1). Additionally, mutations in genes such as ATP13A2, DCTN1, DNAJC6, FBXO7, PLA2G6 and SYNJ1 have been associated with atypical or complex parkinsonism. Furthermore, recent studies have identified several other genes, including CHCHD2, LRP10, TMEM230, UQCRC1 and VPS13C, that are implicated in PD [77,99]. Besides core motor features such as bradykinesia (slowness of movement), rigidity and resting tremor, PD is also associated with a heterogeneous spectrum of nonmotor symptoms that significantly contribute to the overall disease burden of PD [100]. From a clinical perspective, there is no definitive method to diagnose PD in vivo, except for genetic testing in specific circumstances, which is limited to a few cases. Given the protracted timeline over which PD can advance, it imposes significant and far-reaching implications on patients, healthcare providers and society at large [94].

## 3. Mitochondrial Ribosomal Proteins Associated with Mitochondrial Neurodegenerative Diseases

Ribosomes are macromolecular machines with a universally conserved structure and function for protein synthesis. Within the cells of living organisms, mitochondria contain their own ribosomes, known as mitoribosomes, which are primarily responsible for synthesizing essential components of the oxidative phosphorylation machinery [41,101]. Mitoribosomes are located in the organelle matrix and are associated with the inner membrane to facilitate the cotranslational insertion of highly hydrophobic nascent polypeptides [38,102]. In mammals, mtDNA encodes 13 proteins that serve as crucial membrane components of the OXPHOS enzymatic complexes. The mammalian mitoribosome is a 55S ribonucleoprotein complex composed of a 39S large subunit (mt-LSU) containing 52 (MRPs), a 16S rRNA and a structural tRNA (tRNAVal in human cells), as well as a 28S small subunit (mt-SSU) with 30 MRPs and a 12S rRNA. In comparison to bacterial and eukaryotic cytoplasmic ribosomes, the 55S ribosomes contain a lower proportion of RNA, approximately 25–30% [103,104]. All MRPs are encoded in nDNA, synthesized using cytoplasmic ribosomes, and subsequently imported into the mitochondrial matrix. Once inside, they are assembled with subunit-specific RNAs encoded in mtDNA [41,104,105,106,107]. Dysfunctions in mitochondrial translation can lead to severe human diseases and may result from mutations in various components of the mitochondrial translation machinery, including tRNAs, aminoacyl-tRNA synthetases, translation factors and ribosomal components [2,108,109]. Furthermore, mutations or deficiencies in ribosome assembly proteins or other essential proteins can also contribute to mitochondrial diseases, as the mitochondrial ribosome is responsible for translating mRNAs for the 13 essential proteins of the OXPHOS system in affected tissues. Several MRP genes are located in loci associated with disorders related to impaired oxidative phosphorylation, such as Leigh syndrome, multiple mitochondrial dysfunctions and nonsyndromic hearing loss. Therefore, these diseases exhibit genetic heterogeneity and can manifest in a broad spectrum of clinical presentations [37,38,41,107,110,111]. The presence of numerous patients affected by mitochondrial disorders, characterized by multienzymatic OXPHOS defects of unknown genetic origin, suggests that there are still many genes involved in the biogenesis and function of the mitochondrial translation machinery that remain unidentified. Regarding alterations in ribosomal components associated with human diseases, only a limited number of mutations in mitoribosomal proteins have been reported so far [36,38,112]. In recent publications, the focus has been on the detection of pathological mutations in nuclear genes responsible for encoding mitochondrial ribosomal proteins. These findings emphasize the significance of their expression and involvement in mitochondrial translation, thus suggesting their potential role as contributing factors to genetic neurodegenerative diseases in humans [7,16,38,41,112,113,114].

## 4. Three Mitochondrial Ribosomal Proteins as Intermediate Stage in a Path Connected with Alzheimer’s and Parkinson’s

Neurodegenerative disorders are characterized by a gradual and progressive decline, specifically targeting interconnected neuronal systems based on anatomical or physiological relationships [115]. Mitochondria play a critical role in generating the majority of cellular ATP and are indispensable for ensuring optimal neuronal functioning. Dysfunction of mitochondria can give rise to PMDs and potentially contribute to the development of neurodegenerative conditions such as AD and PD. Mitochondria serve as crucial regulators of both cell survival and cell death, exerting a central influence on the aging process, and have been observed to interact with numerous specific proteins associated with genetic forms of neurodegenerative diseases [116]. These diseases include AD, PD, ALS and HD [115,117].

### 4.1. The Pathophysiology of Mitochondrial Diseases

The pathophysiology of mitochondrial diseases is complex and involves genetic mutations in both mtDNA and nDNA. Mitochondrial diseases are typically considered to be disorders caused by biochemical defects in the respiratory chain [117,118]. The regulatory role of nuclear genes in maintaining mitochondrial homeostasis and functionality is now included, along with defects in the lipid milieu, mitochondrial translation and mitochondrial fission and fusion [101,117,118]. Considerable progress has been made in our understanding of the molecular basis of mitochondrial diseases and their genetic etiology, particularly through next-generation sequencing/whole exome sequencing (NGS/WES) approaches. These approaches provide valuable information on the genes implicated in neurodegenerative disorders and could better define the impact of mitochondrial dysfunction on their pathogenesis [119]. As previously mentioned, mitochondrial diseases were caused as a consequence of mutations, whether inherited or spontaneous, in either mtDNA or nDNA. Furthermore, as previously discussed, human cells possess two distinct genomes and two protein synthesis systems. The first genome is the nuclear genome (nDNA), which consists of approximately 3 × 10^9^ base pairs, and a second genome resides within a cytoplasmic organelle known as the mitochondrion (mt). Approximately 1500 nuclear gene products (around 3% of the total) are translated by cytoplasmic ribosomes and subsequently imported into the mitochondrion to perform their functions [120,121]. The cytoplasmic ribosome is composed of four rRNAs (28S, 18S, 5.8S and 5S) and 85 ribosomal proteins. The genes responsible for encoding these components have been mapped and investigated to a considerable extent [122]. Ribosomes play a crucial role as molecular machines, essential for all life on our planet. They decode information carried by messenger RNAs (mRNAs) and translate it into proteins [123]. Ribosome biogenesis is the crucial process in which all the components of ribosomes, such as ribosomal proteins (r-proteins) and rRNAs, come together to form the functional translation machinery. This process involves a large number of proteins, known as maturation factors and RNA factors (such as snoRNAs in cytosolic ribosome maturation) that aid in properly folding, assembling and modifying the different elements of the ribosome [124]. In mitochondria, the process of mitoribosome biogenesis is even more complex due to the need for coordination between two gene expression compartments [125]. Several mutations have been discovered in genes related to the cytoplasmic translation machinery, including ribosome components and numerous interacting factors. These mutations have been linked to various human diseases. On the contrary, genes from both the nuclear and mitochondrial genomes contribute to the formation of mitochondrial ribosomes, also known as mitoribosomes. These mitoribosomes play a crucial role in the synthesis of the oxidative phosphorylation machinery. These genes have been the subject of major research efforts in yeast and humans. Yeast serves as a model system for eukaryotic cell biology, while in humans, mitoribosomes have been implicated in human health [126]. The synthesis of ribosomal proteins occurs on cytoplasmic ribosomes, after which they are imported into the mitochondria. Interference with the synthesis of these proteins and other components of the mitochondrial translation system, such as mitochondrial tRNAs, either through deletion or mutation of the mitochondrial genes, is known to cause various mitochondrial diseases of varying severity. These diseases include myopathies and sensorineural disorders such as blindness and deafness [127]. By analogy, it can be expected that the loss or mutation of any of the 78 proteins required for the function of the mitochondrial ribosome [128] would also result in mitochondrial disease. Published research indicates that certain genes responsible for encoding MRPs are located within chromosomal loci that have already been associated with human disorders, including conditions like deafness, retinitis pigmentosa and Usher syndrome 1E [129]. A number of mutations that affect mitochondrial translation have been identified, including mutations in the core MRPs: MRPS16, MRPS22 and MRPL12 [130]. Additionally, mutations have been found in several accessory proteins, such as ObgH1 and C7orf30 [130]. It appears that mutations in MRPS16 and MRPS22 impact the stability of the mitoribosome [130], while MRPL12 is involved in the recruitment of elongation factors and the modulation of mitochondrial gene expression [129].

### 4.2. The Connection between Mitochondrial Dysfunctions and the Effects of Heavy Metals and Metalloid Oxyanions

Extensive scientific evidence has established a connection between mitochondrial dysfunctions, the harmful effects of heavy metals and metalloid oxyanions and systemic as well as neurodegenerative disorders [131,132]. Specifically, the toxicity of the metalloid tellurium (Te) has been implicated in the etiopathogenesis of various neurodegenerative disorders [132,133]. Accumulated experimental data indicate that tellurium not only exhibits a well-documented garlic-like odor but also exerts significant neurotoxic effects. In the past, Te has been employed as a valuable tool for studying the evolutionary origins of mitochondria in prokaryotes [134]. Recently, a research paper was published focusing on neurodegenerative diseases, utilizing Te as an investigative tool and the yeast S. cerevisiae as a model system [40]. The study employed classical genetics, genomics and molecular biology techniques to examine the influence of three nuclear genes. These genes encoded two MRPs (MRPL44 and NAM9) and one mitochondrial ribosomal biogenesis protein (GEP3). Furthermore, mutations in these three genes have been associated with neurodegenerative diseases such as AD and PD [40]. The importance of this work lies in the utilization of a Te compound, which enabled us to identify an intermediate point. Specifically, this point involves the three proteins of the mitochondrial ribosome, potentially linking damaged genes to neurodegenerative diseases in humans. This study successfully identified three nuclear genes that encode mitochondrial ribosomal proteins. Mutations in these genes result in resistance to Te in the yeast *S. cerevisiae*. Additionally, previous publications have associated the toxic effects of Te with the development of neurodegenerative diseases like AD and PD [132,133]. The recent work by Pontieri et al. [40] represents a significant advancement in scientific knowledge regarding the study of the three MRPs involved in potassium Te resistance in *S. cerevisiae.* It sheds light on their potential role in neurodegenerative dysfunctions such as AD or PD, thus providing valuable insights for further investigation. Future research interests may include studying the physiological function of MRPL44, NAM9 and GEP3 in detail, focusing on their role in mitochondrial translation as well as exploring their potential involvement in other functions apart from translation. This research will contribute to establishing the molecular foundation for comprehending the phenotypes associated with mutations affecting MRPL44, NAM9 and GEP3. Currently, we are examining these mutations in yeast, with the intention of expanding these investigations to other model systems. The ultimate objective is to unravel how genetic defects in these mitochondrial proteins (and others) can lead to neurodegenerative disorders in humans and animals. Additionally, this research aims to pave the way for developing therapeutic drugs to treat these diseases.

## 5. Experimental Hypothesis for the Isolation and Characterization of the Three Proteins MRPL44, NAM9 and GEP3

Elucidating the function of MRPL44, NAM9 and GEP3 (Figure 1) proteins is of paramount importance. For this reason, an experimental strategy aimed at understanding the pathogenic processes associated with these proteins is proposed. The main purpose of the experimental hypothesis is to study the functions, including those not directly related to translation, of three ribosomal mitochondrial proteins, MRPL44, NAM9 and GEP3, in human cells or model systems such as yeast, *Caenorhabditis elegans* (*C. elegans*) or *mice* affected by mitochondrial neurodegenerative disease phenotypes.

The first step is to introduce mutations to the MRPL44, NAM9 and GEP3 genes, using molecular technologies such as systems using CRISPR/CAS9. This technique allows for modifications to be made in the sequence of each of the MRPL44, NAM9 and GEP3 genes in human cells or in model systems such as yeast, *C. elegans* or mice. Subsequently, pure mitochondria from human cells or cells of model organisms can be prepared using the enzymatic protoplastification method or the rapid glass bead milling method. At this point, it would be possible to characterize the mitochondria at the microscopic, biochemical and molecular levels in wild-type and mutant cells of both humans and model systems. For this purpose, different methods can be used, such as the analysis of colocalization by confocal microscopy, the analysis of mitochondrial purity based on marker enzymes such as succinate dehydrogenase, adenylate kinase, fumarase and iso-citrate dehydrogenase, the functional analysis of mitochondria involving the Krebs cycle, the respiratory chain and OXPHOS. The “omics” methodologies such as proteomics, lipidomics, metabolomics and transcriptomics are added to these techniques. It is crucial to analyze the expression of the MRPL44, NAM9 and GEP3 genes in mutated human cell lines or the *C. elegans* Alzheimer’s model expressing the human tau protein. It would be interesting to understand the effect of the mutations in the MRPL44, NAM9 and GEP3 genes on the *C. elegans* Alzheimer’s model. For this purpose, crosses of different C. elegans strains exhibiting these mutations can be performed with the transgenic C. elegans strain harboring the human tau gene, which specifies the AD phenotype.

In addition to in vivo experiments, it is interesting to produce the recombinant MRPL44, NAM9 and GEP3 proteins in yeast to perform biochemical and chemical characterization experiments using mass analysis techniques such as electrospray mass spectrometry (MS), mass spectrometry tandem (MS/MS) and matrix-assisted laser desorption/ionization time-of-flight (MALDI-TOF) mass spectrometry. These methods provide valuable information about the peptide map, the complete amino acid sequence and any post-translational modifications present in each of the aforementioned proteins. The partial results, such as the NH2-terminal sequence, as well as the final results, which encompass the entire protein sequence, obtained from these biochemical procedures, can be utilized for bioinformatic analyses against protein databases like Swiss-Prot, EMBL and others. Research software such as SRS, FASTA and BLASTA can be employed for searching and comparing sequences, while analysis tools like Clustal X can assist in multiple sequence alignment. Finally, antibody production against MRPL44, NAM9 and GEP3 proteins is useful for immunochemical tests such as sandwich enzyme-linked immunosorbent assay (ELISA).

In addition to *C. elegans*, a model for studying these genes could be *S. cerevisiae*. Mutant strains of tellurite-resistant *S. cerevisiae* yeast can be transformed with suitable expression plasmids, in which the sequences of each of the three genes, MRPL44, NAM9, and GEP3, have been cloned to test whether or not this transformation restores wild-type phenotype of tellurite sensitivity. Functional restoration experiments can also be performed in animal models such as *C. elegans* or mice affected by mitochondrial neurodegenerative diseases.

## 6. Conclusions

The identification of gene mutations associated with neurodegenerative diseases is offering valuable insights into the importance of targeted therapies and comprehensive investigations of genetically homogeneous clinical groups. Such endeavors are crucial for accurately delineating the natural progression of these diseases, identifying dependable biomarkers and recognizing long-term changes that occur over extended periods of observation. These could be used as clinical trial endpoints. Numerous instances in the literature document mitochondrial involvement in diverse clinical presentations. Our understanding of the interplay between the nuclear genome, which includes gene products that directly or indirectly function with the mitochondrion and the mitochondrial genome, is expanding considerably. This expansion is largely facilitated by advancements in “omics” disciplines such as genomics, proteomics, metabolomics and more. Attempts have been made to integrate MRPs into this picture by suggesting that their expression and requirement for mitochondrial translation should be considered for possible involvement in clinical conditions. Investigating the association of variants in mitochondrial ribosomal protein (MRP) genes with diverse phenotypes or as genetic modifiers of known gene mutations (e.g., mt-tRNA, mt-rRNA) is crucial. Initially, employing diagnostic in vivo animal models of mitochondrial translation would provide the most comprehensive understanding. Furthermore, it is of the utmost importance to acknowledge the nontranslational roles of MRPs, which encompass potential contributions to selective protein import, facilitating interactions between imported proteins and locally synthesized mitochondrial subunits during the assembly of oxidative phosphorylation (OXPHOS) complexes and exerting translational control over mitochondrial protein synthesis. Researchers are now directing their efforts toward developing therapies that target and safeguard mitochondria and neurons from the toxicity associated with aging and mutant proteins, taking into account the findings of studies that are uncovering the role of mitochondria in disease onset and progression. Tremendous progress is being made in this research and optimism is running high that significant steps can continue, bringing us much closer to a future where devastating diseases like AD and PD can be controlled.

## Data Availability

Not applicable.
